# Salivary non‐apoptotic tumoral microvesicles: A potential progressive marker in oral cancer patients

**DOI:** 10.1111/jcmm.17461

**Published:** 2022-11-29

**Authors:** Qi‐Wen Man, Rui‐Fang Li, Lin‐Lin Bu, Yi Zhao, Bing Liu

**Affiliations:** ^1^ The State Key Laboratory Breeding Base of Basic Science of Stomatology (Hubei‐MOST) & Key Laboratory of Oral Biomedicine Ministry of Education, School & Hospital of Stomatology Wuhan University Wuhan China; ^2^ Department of Oral and Maxillofacial Head Neck Surgery, School & Hospital of Stomatology Wuhan University Wuhan China; ^3^ Department of Prosthodontics, School and Hospital of Stomatology Wuhan University Wuhan China

**Keywords:** EGFR, microvesicles, oral squamous cell carcinoma, saliva

## Abstract

Tumour cell‐secreted microvesicles (MVs) contribute immensely to tumour progression. However, the role of tumoral salivary MVs in oral squamous cell carcinoma (OSCC) remains unclear. Herein, we elucidated the role of non‐apoptotic salivary tumoral MVs in OSCC development, especially relating to the migration ability. We purified and compared non‐apoptotic salivary tumoral MVs from 63 OSCC patients and orthotopic OSCC mice model. Next, we compared the protein difference between apoptotic and non‐apoptotic MVs by Western blot, proteomics and flow cytometry from saliva and CAL27 cells. Finally, we collected the non‐apoptotic MVs and co‐cultured with normal oral epithelial cells, the migration ability was examined by wound healing assay and Western blot assay. Our results indicated that the levels of non‐apoptotic tumoral S‐MVs were significantly higher in OSCC patients with T3 to T4 stages than in patients with T1 to T2 stages or healthy donors. In OSCC mice model, we found elevations of non‐apoptotic tumoral MVs associated with tumoral volume. EGFR overexpression increased the generation of non‐apoptotic tumoral MVs which could significantly promote normal epithelial cell migration. In conclusion, elevated levels of non‐apoptotic tumoral S‐MVs are associated with clinicopathologic features of OSCC patients, implying that non‐apoptotic tumoral S‐MVs are a potential progressive marker of OSCC.

## 1 INTRODUCTION

Oral squamous cell carcinoma (OSCC) is one of the most prevalent oral malignant lesions, accounting for nearly 600,000 new cases every year.^1^ The incidence rate was growing, and most OSCC patients presented with advanced stages had poor outcomes.^2^ Furthermore, the molecular mechanism underlying OSCC development remains largely unknown.^3^ OSCC tissue is immersed in the saliva microenvironment and tumour‐derived proteins or RNAs could be released into the saliva. The biological molecular from saliva could directly be transported to the recipient cells to function in favour of the progression or diagnosis of OSCC.^4^, ^5^

Microvesicles (MVs) are small phospholipid membrane‐enclosed entities which directly released from the membrane in response to stimulation or apoptosis.^6^ Originally, MVs were identified as inert debris, but their functional relevance has become apparent, and their shedding is now recognized as a critical mode of cell‐to‐cell communication.^6^ Previously, study suggested that MVs were associated with apoptosis and characterized using Annexin V, an indicative marker for apoptosis.^6^ Subsequent studies revealed that not all MVs were positive for Annexin V, with Annexin V negative population representing a non‐apoptotic (Annexin V^−^ populations) subtype of MVs.^7^, ^8^ Furthermore, proteomics analysis revealed that non‐apoptotic MVs included unique proteins compared with apoptotic MVs regarding specific signalling pathway activation.^8^ Study suggested a positive correlation between apoptotic MVs and high sensitivity C‐reactive protein levels, a biomarker for inflammation in diseases including diabetes and acute coronary syndrome.^9^ Although non‐apoptotic MVs were linked to poor prognosis in OSCC patients,^10^ their subtypes especially tumoral type and the potential roles in OSCC remain largely unknown.

It was illustrated that the close relationship between tumour‐derived MVs and epidermal growth factor receptor (EGFR) signalling in glioblastoma and breast cancer.^11^, ^12^ EGFR, also known as Her1 or ErbB‐1, is a receptor tyrosine kinase belonging to ErbB family, including ErbB‐2, ErbB‐3 and ErbB‐4.^13^ EGFR amplification was considered a prevalent oncogenic event in OSCC development.^14^ Over 80% of HNSCC cases exhibited an increased membraneous expression of EGFR, and overexpression of receptor is linked to radiotherapy‐resistance.^15^, ^16^ It was shown that EGFR‐containing MVs were present in various body fluids, including saliva, blood and pleural effusions.^17^, ^18^ However, the potential roles of EGFR‐containing MVs, especially the non‐apoptotic EGFR‐containing MVs, in OSCC are largely unknown. Hence, this study aimed to investigate the expression level, releasing mechanism and potential biological effects of non‐apoptotic tumoral S‐MVs in OSCC patients.

## 2 MATERIALS AND METHODS

### 2.1 Patients and sampling

We recruited a consecutive series of 63 Chinese OSCC patients and 24 oral dysplasia patients treated at Hospital of Stomatology, Wuhan University, between May 2016 and August 2017. In addition, 24 healthy donors were included in this study. We excluded 25 OSCC patients for the following reasons: history of previous resection, insufficient pathological evaluation or treated with cetuximab therapy. The age of OSCC ranged from 36 to 83 years old (mean 58 years old). The eighth edition of the American Joint Committee on Cancer TNM staging system was used for TNM classification. The clinicopathologic characteristics of patients are reviewed using the electronic medical record. The use of the clinical samples and the patient data was approved by the review board of the ethics committee of the Hospital of Stomatology, Wuhan University (2016‐A04). The written informed consents were provided from patients or their legal guardians.

### 2.2 Saliva collection

Before saliva collection, the healthy volunteers, oral dysplasia patients and OSCC patients must be forbidden for performing oral hygiene procedures including tooth brushing and gargle, habits such as smoking and alcohol drinking was inhibited. No drinking and eating was allowed for at least 1 h before saliva collecting. An unstimulated saliva sample (5 ml) was collected between 6 AM and 9 AM as previously established.^10^ The saliva was centrifuged at 3000 × *g* for 20 min at room temperature, and the supernatant was then collected and subjected to second centrifugation of 20 min at 3000 × *g* at 4°C to obtain cell‐free saliva, which was stored in aliquots at −80°C.

### 2.3 Isolation of S‐MVs

Frozen saliva samples were thawed at room temperature immediately before MVs isolation and then centrifuged at 3000 × *g* for 20 min at 4°C, and the supernatant was then diluted in PBS with 1:1. The diluted saliva was centrifuged at 20,000 × *g* for 60 min at 4°C. The supernatant was carefully aspirated, and the pellet was resuspended into 150 μl PBS and stored in −80°C.

### 2.4 MVs characterization by transmission electron microscope

Morphological examination of isolated MVs was done using a transmission electron microscope. Typically, 10 μl of isolated MVs were loaded on a 300 mesh copper grid. 2% phosphotungstic acid was stained with MVs for 1–2 min, MV samples were dried with the help of an electric incandescent lamp. The pictures were obtained utilizing a transmission electron microscope (HT7700, Hitachi High‐Tech; Hitachi,) at an accelerating voltage of 160 kV.

### 2.5 MVs and cells characterization by flow cytometry

As previously described,^19^ 10 μl S‐MVs were collected, and 1 μl of antibody was added. Antibodies included FITC labelled anti‐Annexin V (BioLegend,) and PE labelled EGFR (BioLegend,). In addition, Annexin V binding buffer (BioLegend,) was added as the protocol described. The percentages of subtypes of S‐MVs were gated according to the control group (BD LSRFortessa, Wuhan Institute of Virology). For the cellular uptake assay, the PKH67 labelled MVs treated CAL27 cells were collected and tested using FITC channel. The FITC positive group among total cells were gated using Flow Jo software.

### 2.6 S‐MV characterization by immunofluorescent staining

20 μg of s‐MVs were incubated with 10 μM carboxyfluorescein succinimidyl ester (CFSE) (Sigma‐Aldrich,) for 20 min at 37°C. For double immunofluorescent staining, EGFR‐PE and Annexin V‐FITC antibodies at dilution of 1:100 were stained with MVs (10 μg) from CAL27 vector cells and EGFR transfected cells overnight at 4°C. The stained MVs were mounted on the slides. The stained MVs were observed and photographed under the fluorescence microscope (Leica Microsystems).

### 2.7 S‐MVs characterization by nanoparticle tracking analysis

S‐MVs were purified from seven OSCC patients and five healthy individuals. Nanoparticle tracking analysis (NTA) was performed at 28.0°C using a NanoSight NS300 instrument (Malvern Panalytical). All samples were diluted in PBS to a final volume of 1 ml. 140–200 particles/frame was regarded as ideal measurement concentrations. The software settings for MVs were selected. For each measurement, three cycles were performed by scanning 6 cell positions. The concentrations were output as PDF and EXCEL files for analysis.

### 2.8 MVs sorting by flow cytometry

MVs were separated using instrumental cell sorting (100 μm nozzle, FACSAria III, BD Biosciences, Wuhan Institute of Virology), based on their positivity to LCD and negativity to Annexin V‐FITC‐binding (BioLegend,). Droplets containing MVs are negatively charged based on whether MVs has limited the fluorescently tagged antibody. The sorted MVs were re‐centrifuged (20,000 *g* for 40 min) and collected for Western blot assay or flow cytometry assay. For flow cytometry analysis, 5 sorted and unsorted MVs were stained and analysed using the CD8‐PE‐Cy7 (BD Biosciences), EGFR‐PE (BioLegend,) and AXL‐Per CP‐Cy5.5 (BD Biosciences,) antibodies.

### 2.9 Establishment of in vivo xenograft oral squamous cell tumour models

To make orthotopic human oral squamous cell carcinoma xenograft model, human CAL27 cells were cultured in Dulbecco's modified media (DMEM, Hyclone,). The culture medium was with 10% foetal bovine serum (Cell Max,) within cell incubator under 37°C. When CAL27 cells were confluent, the cells were detached from the dish by tryspin (Gibco,). The experimental procedures involving animals were approved by the review board of the ethics committee of the Hospital of Stomatology, Wuhan University. 1*10^6^ cells/ml were collected and injected into the tongue of 4‐6‐week‐old BALB/c nude mice under anesthetization with isoflurane (*n* = 10) (Sigma‐Aldrich,). They were raised under pathogen‐free conditions at SPF animal laboratory centre of School of stomatology, Wuhan University for 2 weeks. Phosphate‐buffered saline (PBS, Servicebio,) injection group was regarded as control group (*n* = 6). 4‐NQO (Sigma‐Aldrich,) was used for the induction of oral cancer at the dilution of 100 μg/mL for 16 weeks (*n* = 20) and then the C57 mice were fed with normal water for another 4 weeks. Among the 4‐NQO‐induced OSCC, 8 mice were confirmed by haematoxylin–eosin staining. Pilocarpine hydrochloride (Sigma‐Aldrich P6503,) was diluted to 0.25 mg/mL in sterile PBS. The saliva was collected after 100 μl pilocarpine intraperitoneal injection of 10 μl/g body under isoflurane anaesthesia. The saliva was collected by 0.5 ml syringe with 29G x ½” needle.

### 2.10 Cell culture and transfection

Human CAL27 cells were cultured in DMEM, and 293 T were cultured in RPMI‐1640 medium, supplemented with FBS (10% [v/v]) (Gibco), penicillin (100 IU), and streptomycin (100 mg/mL) (Bio Basic Inc.,). The amplified cDNA for EGFR‐EGFP was inserted into pcDNA3.2/V5/GW/D‐TOPO (Invitrogen), and the plasmid DNAs were employed to transfect 293 T cells with Lipofectamine 2000 (Invitrogen,). The supernatant was collected and co‐cultured with CAL27 cells with different concentrations. As a control, the enhanced green fluorescent protein (EGFP) gene in pcDNA3.2 was deployed to transfect CAL27 cells. After co‐culture with lentivirus particles from supernatant of 293 T cells for 72 h, the transfected CAL27 cells were collected and examined for the EGFR expressions by Western blot assay.

### 2.11 Western blot

Proteins from EGFR‐overexpressed CAL27, and corresponding vector cells, also the MVs from the cells were collected. In addition, 20 μg protein of Annexin V^−^ sorted or unsorted MVs were prepared. The protein concentrations were calculated by bicinchoninic acid assay (Pierce BCA, Thermo Fisher Scientific,). Proteins were denatured by adding 5× sodium dodecyl sulphate–polyacrylamide gel electrophoresis (SDS‐PAGE) buffer (Servicebio,) and transferred in PVDF (polyvinylidene fluoride) membrane. The transferred membranes was incubated with EGFR (1:1000, Cell signaling technology), CD8 (1:1000, Proteintech), AXL (1:1000, Cell signaling technology), and GAPDH (1:1000, Proteintech) overnight at 4°C. Then, the membranes were incubated with the corresponding second antibodies for 1 h and washed by TBST (0.1% Tween in TBS). Finally, the membranes were incubated with ECL mix (prepared by ECL A and ECL B) for 2 min and visualize the result in the dark room.

### 2.12 Liquid chromatography (LC)‐electrospray ionization (ESI) tandem mass‐spectroscopy (MS/MS) Analysis

After characterization, a total of 50 μg of protein was used for proteomic analysis using for each of the groups (*n* = 2, for CAL27 Vector and CAL27 EGFR‐OE‐derived MVs, respectively). The following LC–MS analysis was performed by Wuhan Servicebio Technology Co., Ltd, Wuhan, China. Briefly, we use trypsin to digest the precipitated MV samples. The resulting peptides were analysed on an Ultimate 3000 RSLCnano‐UHPLC system connected to a Q Exactive mass spectrometer (Thermo Fisher Scientific,) equipped with a nano‐electrospray ion source. The reference database (SwissProt database, Human) was used for peptide identification and the multiple identified. Protein was identified and validated, using Scaffold_4.8.7 (Proteome Software Inc).

### 2.13 MVs uptake assay

Normal oral epithelial cells were stained with the CellTrace™ CFSE Cell Proliferation Kit (Invitrogen,) according to the manufacturer's instructions. The PKH67 Fluorescent Cell Linker Kit (Sigma) was used to label CAL27 cell‐derived MVs. PKH67 dye solution (1:1000) was mixed with 10 μg or 50 μg MVs for 20 min, washed with PBS, and centrifuged at 20,000 × *g* at 4°C for 60 min. Then, PKH67‐labelled MVs were added to the DIL‐labelled cells and co‐cultured for 24 h. Uptake of MVs at different time points was observed and captured by a confocal fluorescence microscope and flow cytometry.

### 2.14 Wound healing assay

For wound‐healing experiment, cells grew to 80% confluence in six‐well plates and were scratched by sterile 200 μl pipette tips. The wound closure was observed and was imaged under a microscope at 0 and 48 h after MVs treatment (20 μg/mL). The area of un‐adherent or total area was calculated by Image J software and compared by GraphPad software.

### 2.15 Immunohistochemistry

OSCC tissue from xenograft tumour models were collected and formalin‐fixed for immunohistochemistry. Briefly, tissue sections were deparaffinized using xylene and rehydrated in PBS. Citrate solution (Servicebio,) was used for antigen retrieval. The slides contained citrate solution was heated to 125°C and sustained for 8 min and then slides were cooling to 90°C for 1 min. Then, the slides were carried out in the room temperature for 2 h. 3% hydrogen peroxide was incubated with slides for 20 min. Goat serum (Zhongshan golden bridge,) was used for non‐specific binding blocking for 10 min. EGFR antibody at a dilution of 1:200 (Cell signaling technology, 1:200) were incubated for slides overnight at 4°C. After washing in PBS, the secondary antibody (Abbkine, goat‐anti‐rabbit, 1:200) was incubated with the slides for 15 min at room temperature. The slides were incubated with DAB for 30 s. Finally, slides were stained with haematoxylin for visualization. Then, slides were dehydrated using ethanol and cover slipped by thin glass for detection.

### 2.16 Statistical analyses

Sample size was performed with PASS2021 software (NCSS LLC.,). A prior sample size calculation for 3 groups (i.e. healthy donors VS. oral dysplasia and OSCC) using One‐Way Analysis of Varianc F‐Tests assuming a 5% type 1 error (i.e. rejecting a true null hypothesis) rate, 20% type 2 error (i.e. not rejecting a false null hypothesis; 90% statistical power). The proportions of Annexin V^−^/EGFR^+^ S‐MVs were achieved in the pre‐test and the results showed 14.2% ± 8%, 15.8% ± 7% and 22.5% ± 8%. A one‐way anova study with a sample of 54 subjects divided among 3 groups, achieves a power of 92%. This power assumes a non‐central *F* test with a significance level of 0.05. Assuming the drop rate was 20%, total 68 patients should be enrolled in the study. The group subject counts are 23, 23, 23, respectively. All data are presented as mean ± SD of at least three independent experiments. All statistical analyses were performed with GraphPad Prism 6.0 and data comparison between two groups was analysed by the Student's t‐test. Correlation analysis was conducted by the Spearman method. *p* < 0.05 was considered significant.

## 3 RESULTS

### 3.1 Clinical characteristics of OSCC patients

In this study, the age distribution and gender ratio were similar among healthy donors (*n* = 24), oral dysplasia patients (*n* = 24) and OSCC patients (*n* = 63), without statistically significant difference. In addition, none of OSCC patients received anti‐EGFR mono‐antibody therapy before collecting the salivary MVs. Among the OSCC patients, 68.3% was T1 and T2 stages and 60.3% patients without lymph node invasion or distant metastasis. 63.4% OSCC patients were treated with surgery alone and 36.6% OSCC patients were treated surgery plus radiation (Table 1).

**TABLE 1:** Clinicopathologic parameters of OSCC patients included in the study

Clinicopathologic data	Patients (*n* = 63) (%)
Age, Y	
≤58	33 (52.4)
>58	30 (47.6)
(Range, 36–83)	
Gender	
Male	47 (74.6)
Female	16 (25.4)
Tumour stage	
T1 + T2	43 (68.3)
T3 + T4	20 (31.7)
Nodal stage	
NO	38 (60.3)
N1 + N2	25 (39.7)
UICC stage	
I + II	40 (63.4)
III + *IV*	23 (36.6)
Distant metastasis	
M0	63 (100)
Treatment	
Surgery	40 (63.4)
Surgery+ radiotherapy	23 (36.6)

### 3.2 The basic characteristics of S‐MVs in OSCC patients

**FIGURE 1:**
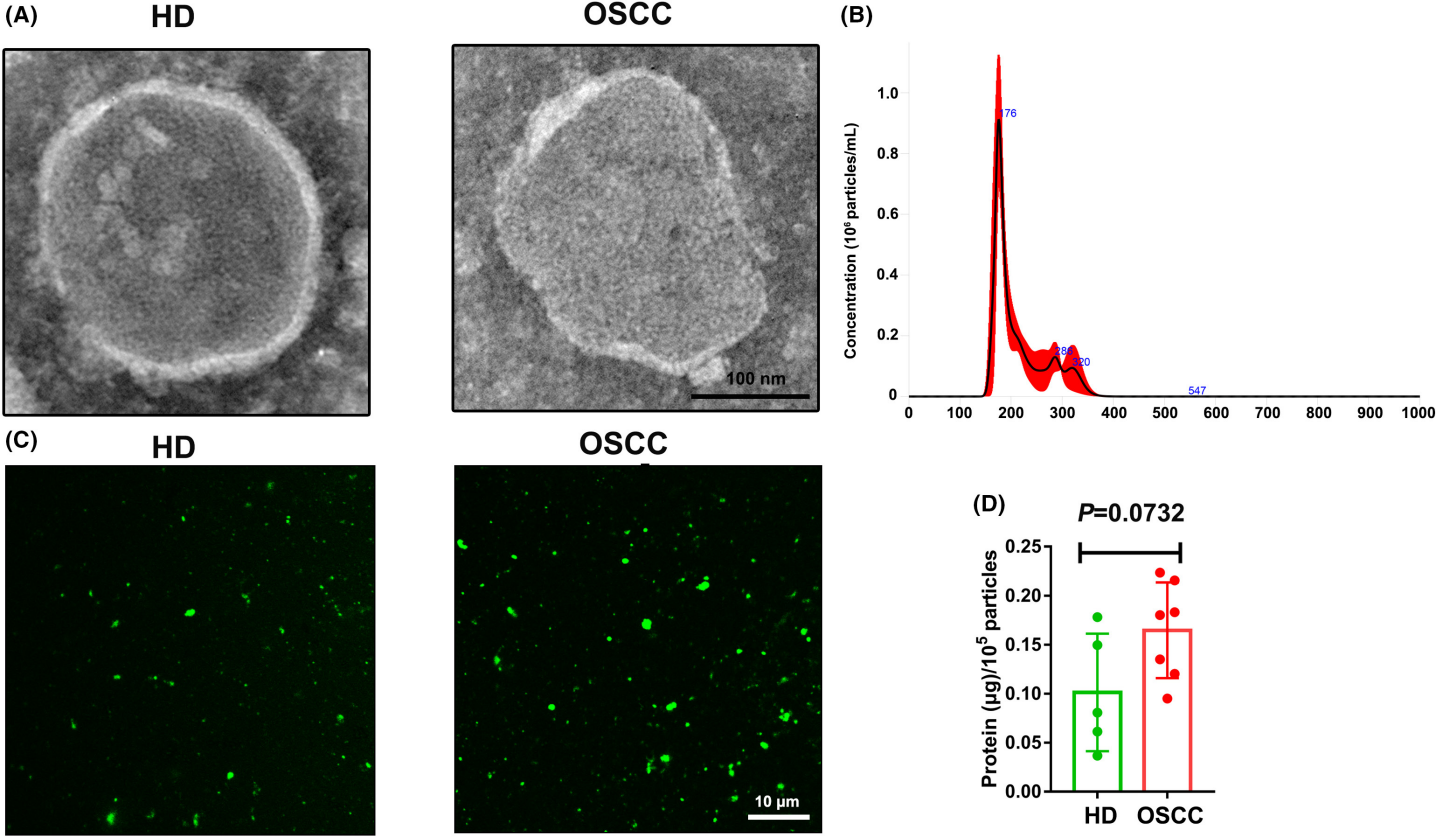
Basic characteristics of salivary microvesicles (S‐MVs) collected from healthy donors and oral squamous cell carcinoma (OSCC) patients. A. Representative images of transmission electron microscope for S‐MVs. B. Statistical analysis of the diameters of S‐MVs by nanoparticle tracking analysis. C. Carboxyfluorescein succinimidyl ester (CFSE) staining of S‐MVs for healthy donors and OSCC patients. D. Nanoparticle tracking analysis of the protein concentrations per 10^5^ particles of S‐MVs from healthy donors (*n* = 5) and OSCC patients (*n* = 7)

S‐MVs were purified from healthy donors and OSCC patients which showed a membranous structure with a diameter about 200 nm, as revealed by transmission electron microscope images (Figure 1A). By nanoparticle tracking analysis, the diameter of collected MVs were calculated, we also found that S‐MVs had a size range within 1000 nm, with mostly nearly 200 nm in diameter (Figure 1B). Additionally, carboxyfluorescein diacetate succinimidyl ester (CFSE) labelling assay demonstrated that CFSE could successfully label purified S‐MVs, indicating their membrane‐bound structures (Figure 1C). The protein concentrations per 10^5^ particles of S‐MVs in OSCC patients (*n* = 7) were slightly higher than that in healthy individuals but without significance (*n* = 5, *p* = 0.0732) (Figure 1D). The above data reveal that purified S‐MVs are consistent with the basic features of MVs and previous report.^10^

### 3.3 Increased non‐apoptotic tumoral S‐MVs are correlated with clinicopathologic features of OSCC patients

**FIGURE 2:**
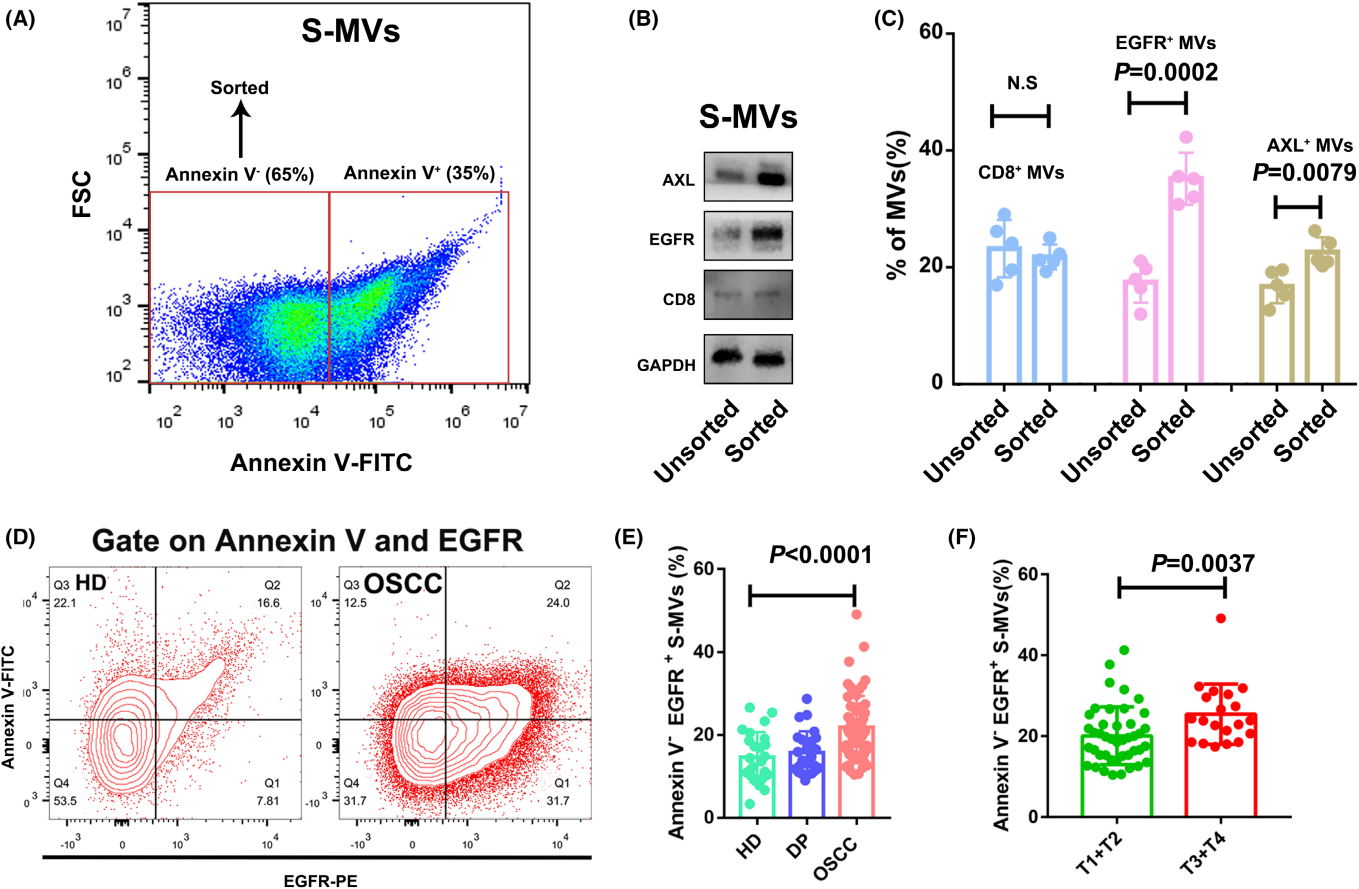
Clinical significance of non‐apoptotic tumour‐derived S‐MVs in oral squamous cell carcinoma (OSCC) patients. A. Representative flow cytometric image of Annexin V staining in S‐MVs. B. Western blot assay revealed that EGFR, AXL was increased in the Annexin V^−^ sorted MVs group. C. Flow cytometric analysis revealed that EGFR^+^ and AXL^+^ MVs were increased in the Annexin V^−^ sorted group. D‐E. Flow cytometric analysis revealed that non‐apoptotic tumoral MVs (Annexin V^−^/EGFR^+^ MVs) were increased in the OSCC group compared with healthy donors. F. Annexin V^−^/EGFR^+^ MVs were significantly upregulated in OSCC patients with late T stages

Given that large proportions of S‐MVs were negative for Annexin V, an apoptotic marker, we employed FACS Aria Cell Sorter to sort Annexin V negative populations (Figure 2 A). After sorting, we analysed the sorted MVs and tested them for Annexin V, which proved to be negative (Figure S1). To identify potentially different proteins in apoptotic and non‐apoptotic MVs, we utilized a Western blot assay to compare the proteins in sorted (Annexin V^−^ population, non‐apoptotic population) and unsorted MVs. The results indicated that EGFR and AXL protein was significantly upregulated in sorted S‐MVs (Annexin V^−^ population, non‐apoptotic population) compared with unsorted S‐MVs (Figure 2B). In addition, the flow cytometric analysis revealed that the level of EGFR and AXL protein in sorted salivary MVs (Annexin V^−^ population, non‐apoptotic population) was significantly higher than unsorted MVs (*n* = 5) (Figure 2C, Figure S2). The above results indicated that tumour‐promoting proteins were elevated in non‐apoptotic S‐MVs compared with total S‐MVs. Then, by examining non‐apoptotic tumoral S‐MVs in OSCC patients and healthy donors, we found that the ratio of Annexin V^−^/EGFR^+^ MVs were significantly upregulated in OSCC patients (Figure 2D, E) and was strongly linked to their T stages (*P* = 0.0037, Figure 2F). All these results indicated that non‐apoptotic tumoral MVs might be closely related to in vivo OSCC development.

### 3.4 Increased non‐apoptotic tumoral S‐MVs are correlated with clinicopathologic features of OSCC in mice model

**FIGURE 3:**
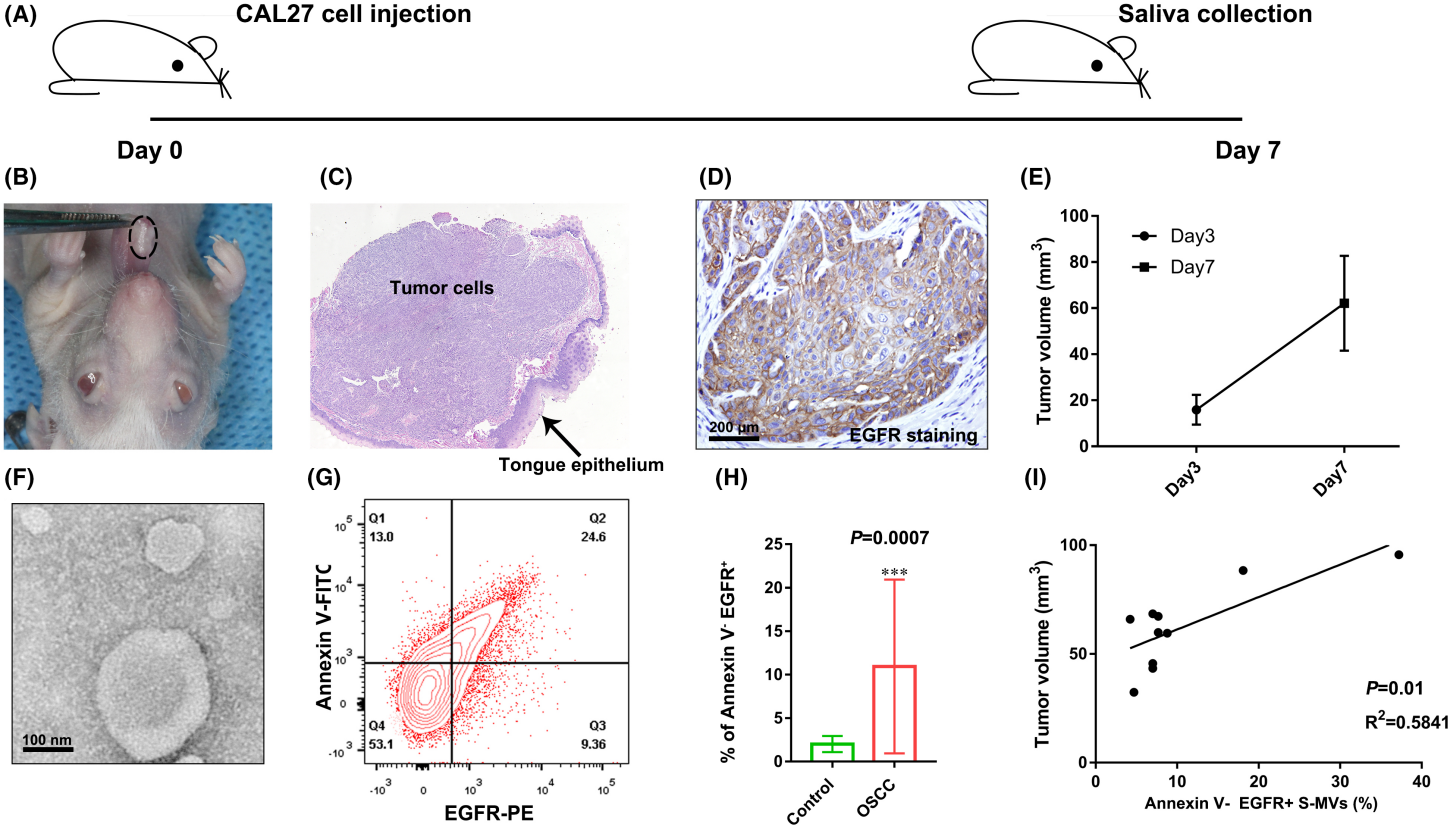
Clinical significance of non‐apoptotic tumoral S‐MVs in oral squamous cell carcinoma (OSCC) mice models. A. Flowchart of saliva collection from OSCC mice model. B‐D. Representative photos, haematoxylin and EGFR staining images of tongue OSCC after injection of CAL27 cells for 7 days. E. Growth curve of tumour after CAL27 cell injection for 3 days and 7 days. F. Transmission electron microscope of collected salivary MVs. G. Flow cytometric analysis of non‐apoptotic tumoral (Annexin V^−^/EGFR^+^) S‐MVs collected from saliva of OSCC. H. Comparison of salivary non‐apoptotic tumoral MVs (Annexin V^−^/EGFR^+^) in control and OSCC group. I. Correlation analysis of tumoral volume and Annexin V^−^/EGFR^+^ levels in S‐MVs

To further validate the preceding results, we generated an OSCC mice model by injecting CAL27 cells into the tongues of nude mice. Firstly, we injected cultured CAL27 cells (1 x 10^6^ cells/tongue) into the tongues of nude mice and collected saliva after 7 days (Figure 3A,B). Next, we employed haematoxylin and human EGFR antibody to detect tumour cells within tongue tissue and discovered that injected tumours had apparent EGFR membrane staining (Figure 3C,D). The tumoral volume was calculated and represented in the growth curve (Figure 3E). After pilocarpine stimulation, we collected saliva from mice and ultracentrifuged for MVs collection. The transmission electron microscope of collected salivary MVs were shown in Figure 3F. Flow cytometric analysis revealed that Annexin V^−^/EGFR^+^ proportions (human EGFR) were mostly below 10% and significantly upregulated in OSCC group (*p* = 0.0007, Figure 3G, H), but were closely associated with tumoral volume (*n* = 10) (*p* = 0.01, *R* ^2^ = 0.584, Figure 3I). In addition, we employed 4‐nitroquinoline N‐oxide‐ (4‐NQO) induced OSCC mice and collected saliva and also found significantly high proportions of Annexin V^−^/EGFR^+^ S‐MVs in 4‐NQO‐induced OSCC group (Figure S3). These findings suggested that non‐apoptotic tumoral MVs (Annexin V^−^/EGFR^+^) indicated a close association with tumour growth in vivo.

### 3.5 Non‐apoptotic tumour cell‐derived MVs secretion is related with EGFR activation

**FIGURE 4:**
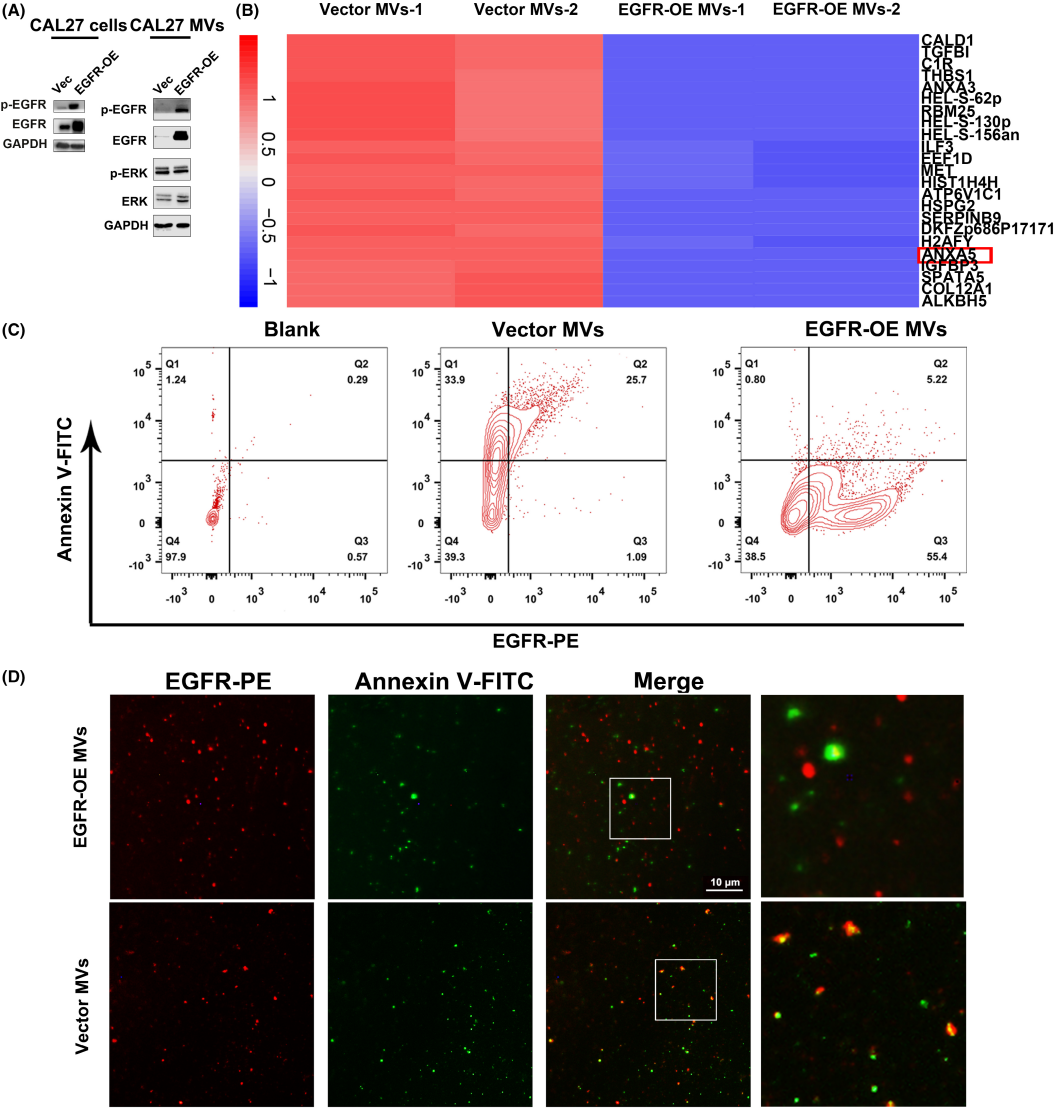
EGFR activation leads to non‐apoptotic tumour‐derived MVs in CAL27 cells. A. Western blot assay revealed that EGFR transfection results in EGFR signalling pathway activation in CAL27 cells and corresponding MVs. B. Mass spectrometry analysis suggested that Annexin V was down‐regulated in EGFR transfected CAL27 cells‐derived MVs compared with vector cells‐derived MVs (red box). C. Flow cytometric analysis indicated that Annexin V^−^/EGFR^+^ MVs were enriched in EGFR transfected group‐derived MVs. D. Immunofluorescent staining result also suggested that Annexin V was less frequently co‐expressed with EGFR in EGFR transfected CAL27 cell‐derived MVs compared with vector cell‐derived MVs

To explore the various proteins involved in apoptotic and non‐apoptotic tumoral MVs release in OSCC, we constructed an EGFR overexpression cell line in CAL27 cells (Figure 4A). First, we explored EGFR, p‐EGFR, ERK and pERK proteins in EGFR transfected cells, corresponding vector cells and cell released MVs. The Western blot assay revealed that EGFR and p‐EGFR was significantly upregulated in EGFR transfected cell‐derived MVs (Figure 4A). Following that, we collected MVs from vector cells and EGFR transfected CAL27 cells, and the proteome analysis revealed that MVs from EGFR transfected CAL27 cells demonstrated significantly lower expression of Annexin V compared with vector cells‐derived MVs (Figure 4B). Using flow cytometric analysis, we subsequently evidenced that Annexin V^−^/EGFR^+^ MVs were enriched in EGFR transfected cell‐derived MVs group (Figure 4C). The immunofluorescence staining of EGFR and Annexin V in cells‐derived MVs also revealed that EGFR transfected cells‐derived MVs showed rare colocalization of EGFR and Annexin V (Figure 4D). These findings demonstrate that the EGFR signalling pathway‐activated cells are closely linked to the releasing of non‐apoptotic tumoral MVs.

### 3.6 Non‐apoptotic tumoral cell‐derived MVs promote normal epithelial cells invasion

**FIGURE 5:**
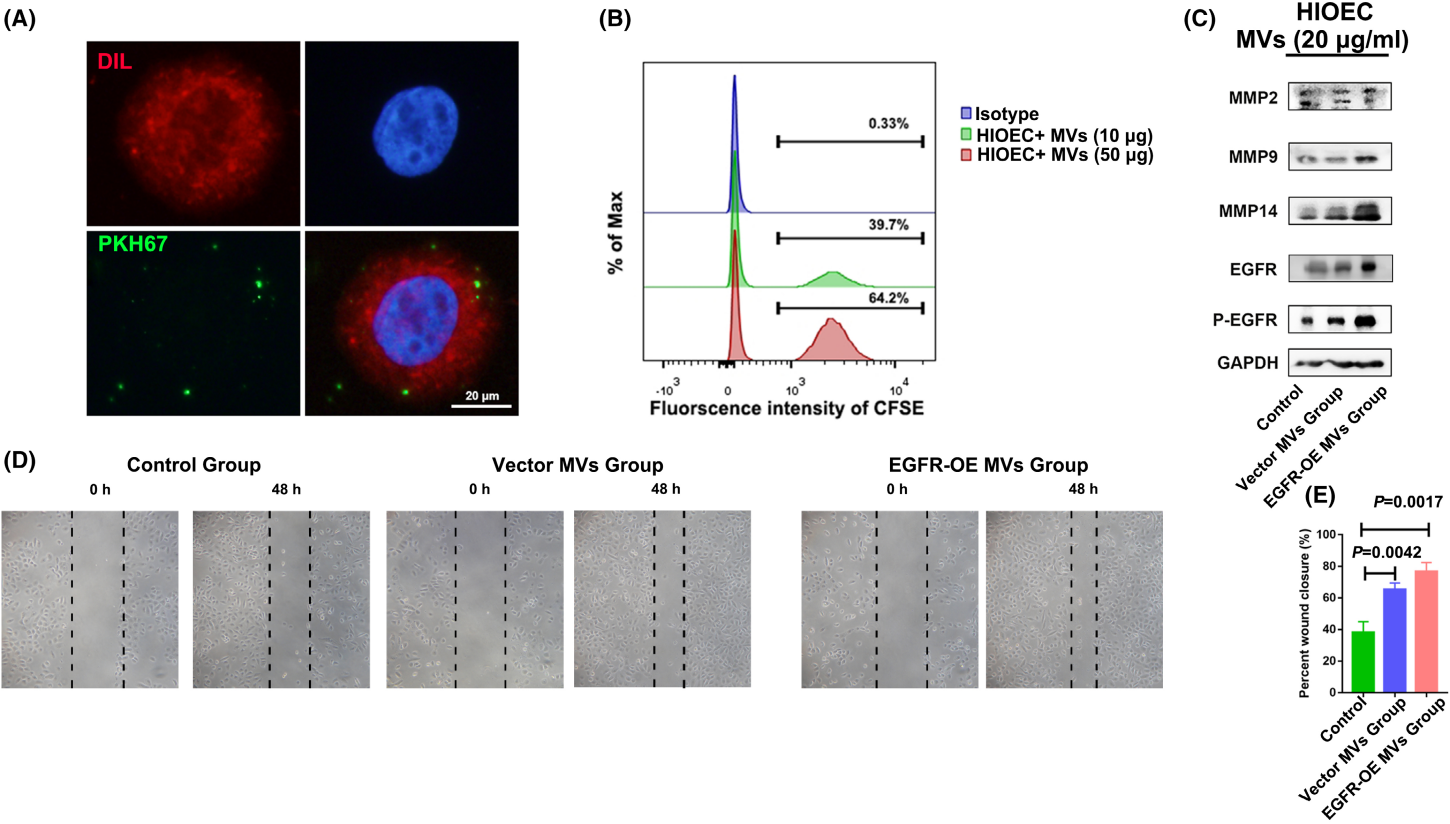
Non‐apoptotic tumoral MVs boost normal oral epithelial migration. A. Uptake assay revealed that MVs were taken up by normal oral epithelial cells. B. Flow cytometric analysis revealed that MVs could be detected in the membrane of normal oral epithelial cells. C. Western blot assay revealed that MMPs including p‐EGFR, MMP9 and MMP14 were upregulated in non‐apoptotic MVs treated normal epithelial cells. D‐E. Migration assay indicated that non‐apoptotic MVs treated normal epithelial cells revealed increased migration ability

To further elucidate the biological significance of non‐apoptotic tumoral MVs, non‐apoptotic tumoral MVs from EGFR transfected CAL27 cells were added to human immortal oral epithelial cells (HIOECs). MVs uptake assay revealed that non‐apoptotic tumoral MVs could be taken up by HIOECs (Figure 5A,B). The Western blot assay also revealed that non‐apoptotic tumoral MVs significantly upregulated p‐EGFR, MMP‐9 and MMP‐14 proteins (Figure 5C). In addition, wound healing assay revealed that non‐apoptotic tumoral MVs significantly increase the migration ability of HIOECs (*p* = 0.0017, EGFR‐OE MVs group VS Control Group) (Figure 5D). These findings imply that non‐apoptotic tumoral MVs might promote the invasion of OSCC by activating EGFR‐MMPs signalling pathway.

## 4 DISCUSSION

Recently, it was indicated that the level of circulating MVs were higher in OSCC patients and were closely connected with increased procoagulant activity and tumour angiogenesis.^19^, ^20^ In addition, in poorly differentiated OSCC patients, the ratio of Annexin V^+^ to Annexin V^−^ S‐MVs, which represented the ratio of apoptotic S‐MVs and non‐apoptotic S‐MVs, was significantly decreased.^10^ Although this study demonstrated the existence of non‐apoptotic MVs in saliva of healthy donors, the link between non‐apoptotic tumoral MVs and OSCC development remains uncertain.^10^ Herein, we found that the level of non‐apoptotic tumoral S‐MVs is upregulated in OSCC patients and demonstrates a close correlation with T stages. More importantly, our study revealed that CAL27 cells with EGFR signalling activation release more non‐apoptotic tumoral MVs, which could significantly promote normal epithelial cell invasion via increased matrix metalloproteinases.

It has been widely studied that cell apoptosis or activation could release MVs with varying contents and effects.^6^, ^7^, ^8^, ^21^ Extensive plasma membrane blebbing and nuclear fragmentation lead to the formation of apoptotic bodies engulfed and removed sequentially by phagocytic cells to prevent spillover and damage to the surrounding cells or tissues.^8^ Apoptotic MVs (200–1000 nm in diameter) are thought to be produced by plasma membrane budding in apoptotic processes.^22^ Apoptotic MVs could induce proinflammatory cytokines through the transfer of their cargo to recipient immune cells or to suppress the immune system.^8^ In systemic lupus erythematosus patients, circulating microparticles were highly positive for Annexin V, and the apoptotic cell‐derived MVs or microparticles had proinflammatory effects on dendritic cells and induced cytokine release.^21^ However, the non‐apoptotic cells (Annexin V^−^ populations) also could release MVs which showed significant different concentrations and contents compared with apoptotic MVs.^22^

Oral cancers were directly immersed in the saliva environment and tumour‐derived RNAs or proteins could be released into saliva which were proved to be involved in the diagnosis or mechanism of OSCC.^23^, ^24^, ^25^ The human epidermal growth factor receptor (EGFR) is an extensively studied oncogenic gene influencing tumorigenesis including proliferation, angiogenesis and metastasis. EGFR had been proved to be overexpressed in over 80% of head and neck tumours and the association translates to shorter survival for patients.^15^ EGFR had also been shown to be packaged into MVs and function as long messengers which are vital for angiogenesis‐modulating factors.^26^ In this study, we identified that EGFR was highly enriched in Annexin V^−^ S‐MVs (non‐apoptotic populations), and showed a close relation with T stages of OSCC patients. In OSCC mice models, they also indicated the potential regulatory mechanism of Annexin V^−^ tumoral S‐MVs in OSCC development.

Annexin V had been regarded as a common marker for total MVs, but subsequent study revealed that only a small proportion was Annexin V^+^ in MVs which represented as an apoptotic subpopulation.^7^ EGFR overexpression had been proved to be related with the EGF‐induced transcripts.^27^ EGFR signalling pathway activation, achieved by EGFR transfection in CAL27 cells, also could lead to the non‐apoptotic tumoral MVs (Annexin V^−^ /EGFR^+^) release evidenced by flow cytometry and proteomics analysis. These result linked EGFR signalling pathway activation with non‐apoptotic tumoral MVs biogenesis. Although, we evidenced that EGFR activation induced by EGFR transfection could significantly induce non‐apoptotic MVs release. However, the precise sorting mechanism of Annexin V in EGFR‐activated cells remain to be further revealed. Especially the endosomal sorting complex required for transport‐related proteins relating to non‐apoptotic MVs release are in urgent need to be further characterized.

MMP expression has been observed to be modulated by EGFR interaction with its ligands.^28^, ^29^, ^30^ Interaction of EGFR with its ligands has been reported to modulate MMP expressions.^28^, ^29^, ^30^ In ovarian cancers, activation of EGFR activation stimulates signalling cascades which could lead to increased expression of MMP‐9 and MT1‐MMP.^28^ EGFR activation has also been reported to induce MMP‐9 expression in bladder cancers.^29^ In hepatocyte growth factor factor‐stimulated keratocytes, researchers found stated significant elevations of MMP‐9 elevations.^31^ Non‐invasive saliva‐based EGFR gene mutation and activated EGFR signalling pathway had been tested in patients with lung cancer.^32^ Herein, we have shown that the non‐apoptotic tumoral MVs from EGFR activated CAL27 cells‐derived MVs could lead to the activation of EGFR signalling pathway in oral epithelial cells. And subsequent elevation of MMPs in these oral epithelial cells which was further evidenced by functional experiment of wound healing assay.

In conclusion, we have identified elevated non‐apoptotic tumoral MVs in OSCC and demonstrated a close relation between non‐apoptotic tumoral MVs and the progression of OSCC in relation to EGFR signalling activation. The non‐apoptotic tumoral MVs is a promising progressive marker for OSCC.

## AUTHOR CONTRIBUTIONS

**Qi‐Wen Man:** Formal analysis (lead); funding acquisition (equal); investigation (lead); writing – original draft (lead). **Rui‐Fang Li:** Funding acquisition (equal); investigation (lead). **Lin‐Lin Bu:** Methodology (lead); validation (lead). **Yi Zhao:** Conceptualization (lead); data curation (equal); resources (lead); writing – review and editing (lead). **Bing Liu:** Data curation (lead); funding acquisition (equal); supervision (lead); validation (lead).

## CONFLICT OF INTEREST

The authors declare that they have no conflicts of interest.

## Supporting information


FigureS1‐S3

## Data Availability

The data analyzed in the present study are available from the corresponding author on reasonable request.
