# Supplementary material for: Salivary non‐apoptotic tumoral microvesicles: A potential progressive marker in oral cancer patients
Source: J Cell Mol Med. 2022 Nov 29;26(24):5955–65. doi: 10.1111/jcmm.17461 (PMC9753445; doi:10.1111/jcmm.17461)
Supplement: Supplementary file 1 — FigureS1‐S3 [file JCMM-26-5955-s001.docx]

**Supporting Information**

**Supplementary Figures**


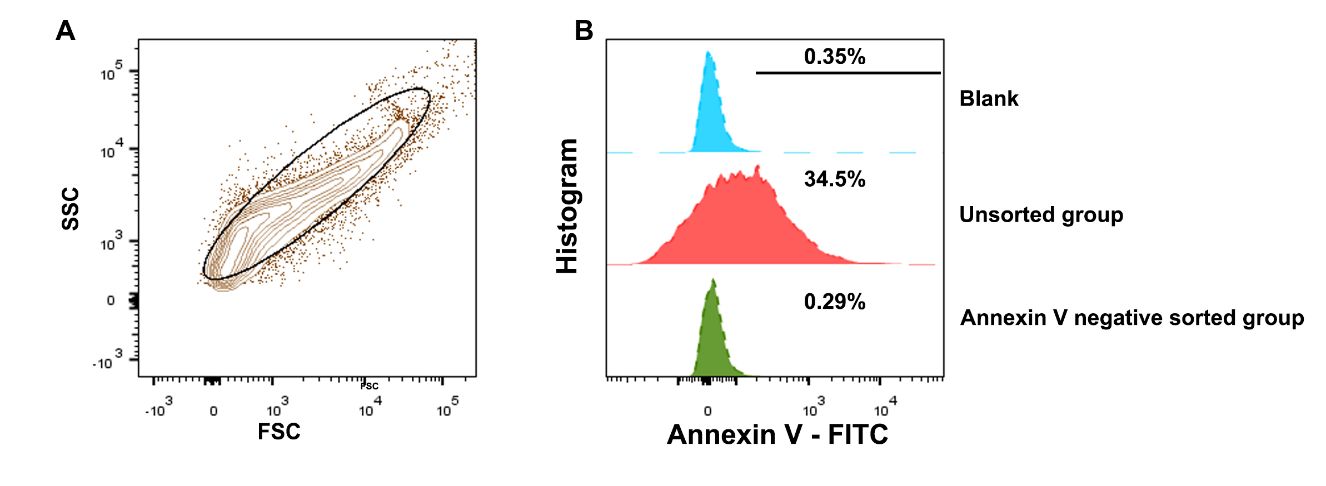


**Fig. S1.** A. Represent flow cytometric plot of salivary microvesicles (MVs) from oral squamous cell carcinoma patients; B. Flow cytometric analysis of Annexin V staining in unsorted group and Annexin V negative sorted group.


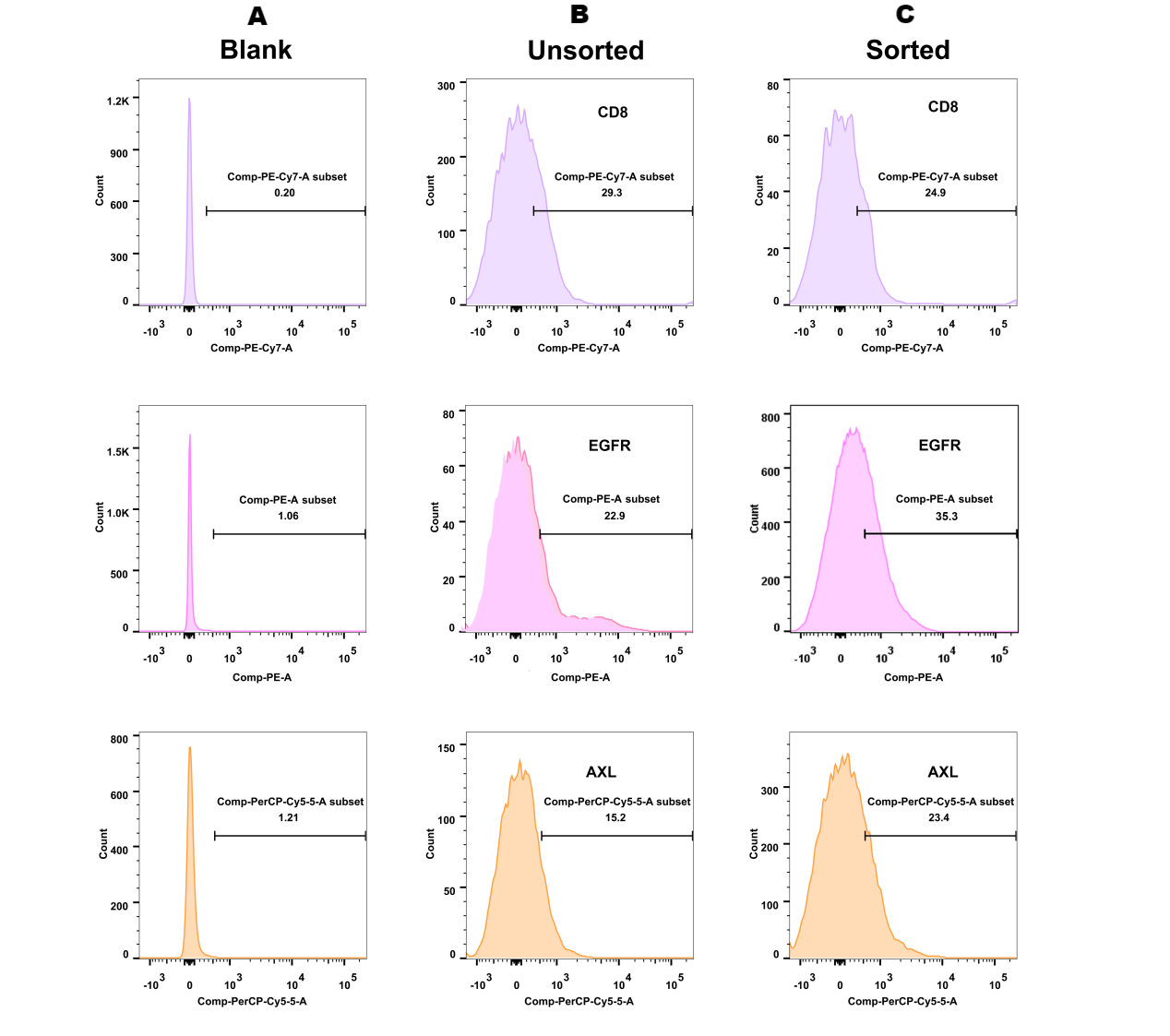


**Fig. S2.** Representative flow cytometric images of expression of cellular markers (CD8, EGFR, and AXL) in unsorted group and Annexin V negative sorted group (A-C).


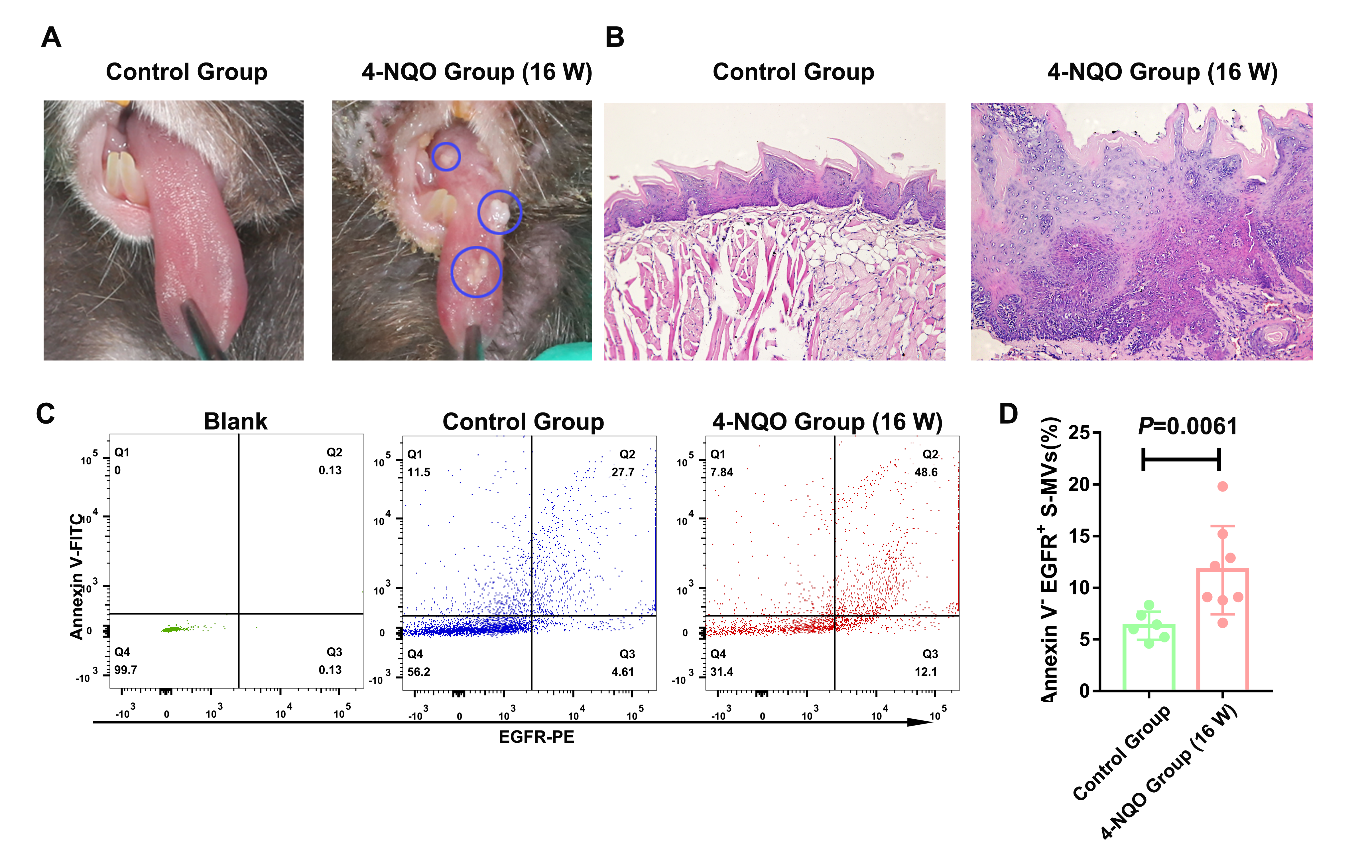


**Fig. S3.** A. Photograph of togue from normal C57 mice and 4-NQO induced carcinogenesis of C57 mice; B. Hematoxylin-eosin staining of tongue tissue from normal C57 mice and 4-NQO induced carcinogenesis of C57 mice after 16 weeks induction; C. Flow cytometric images of Annexin V^-^/EGFR^+^ expression in salivary MVs from normal C57 mice and 4-NQO induced carcinogenesis of C57 mice; D. Comparison of the levels of Annexin V^-^/ EGFR^+^ MVs in normal C57 mice (n=6) and 4-NQO induced carcinogenesis of C57 mice (n=8).

**Supplementary Material and Methods**

**S-MVs characterization by nanoparticle tracking analysis**

S-MVs were purified from seven OSCC patients and five healthy individuals. Nanoparticle tracking analysis (NTA) was performed at 28.0 °C using a NanoSight NS300 instrument (Malvern Panalytical). All samples were diluted in PBS to a final volume of 1 mL. 140–200 particles/frame was regarded as ideal measurement concentrations. The software settings for MVs were selected. For each measurement, three cycles were performed by scanning 6 cell positions. The concentrations were output as PDF and EXCEL files for analysis.

**MVs sorting by flow cytometry**

MVs were separated using instrumental cell sorting (100 μm nozzle, FACSAria III, BD Biosciences, Wuhan Institute of Virology), based on their positivity to LCD and negativity to Annexin V-FITC-binding (BioLegend, USA). Droplets containing MVs are negatively charged based on whether MVs has limited the fluorescently-tagged antibody. The sorted MVs were re-centrifuged (20,000 g for 40 min) and collected for western blot assay or flow cytometry assay. For flow cytometry analysis, 5 sorted and unsorted MVs were stained and analyzed using the CD8-PE-Cy7 (BD Biosciences, USA), EGFR-PE (BioLegend, USA) and AXL-Per CP-Cy5.5 (BD Biosciences, USA) antibodies.

**Establishment of in vivo xenograft oral squamous cell tumor models**

To make orthotopic human oral squamous cell carcinoma xenograft model, human CAL27 cells were cultured in Dulbecco’s modified media (DMEM, Hyclone, USA). The culture medium was with 10% fetal bovine serum (Cell Max, China) within cell incubator under 37°C. When CAL27 cells were confluent, the cells were detached from the dish by tryspin (Gibco, USA). The experimental procedures involving animals were approved by the review board of the ethics committee of the Hospital of Stomatology, Wuhan University. 1*10^6^ cells/ml were collected and injected into the tongue of 4- to 6-week-old BALB/c nude mice under anesthetization with isoflurane (n=10) (Sigma-Aldrich, St Louis, MO, USA). They were raised under pathogen-free conditions at SPF animal laboratory center of School of stomatology, Wuhan University for 2 weeks. Phosphate-buffered saline (PBS, Servicebio, Wuhan, China) injection group was regarded as control group (n=6). 4-NQO (Sigma-Aldrich, St Louis, MO, USA) was used for the induction of oral cancer at the dilution of 100 μg/ml for 16 weeks (n=20) and then the C57 mice were fed with normal water for another 4 weeks. Among the 4-NQO induced OSCC, 8 mice were confirmed by hematoxylin-eosin staining. Pilocarpine hydrochloride (Sigma-Aldrich P6503, St Louis, MO, USA) was diluted to 0.25 mg/ml in sterile PBS. The saliva were collected after 100 μL pilocarpine intraperitoneal injection of 10 µL/g body under isoflurane anesthesia. The saliva was collected by 0.5 mL syringe with 29G x ½" needle.

**Cell culture and transfection**

Human CAL27 cells were cultured in DMEM, and 293T were cultured in RPMI-1640 medium, supplemented with FBS (10% (v/v)) (Gibco, USA), penicillin (100 IU), and streptomycin (100 mg/mL) (Bio Basic Inc., Shanghai, China). The amplified cDNA for EGFR-EGFP was inserted into pcDNA3.2/V5/GW/D-TOPO (Invitrogen, Carlsbad, CA, USA), and the plasmid DNAs were employed to transfect 293T cells with Lipofectamine 2000 (Invitrogen, USA). The supernatant was collected and co-cultured with CAL27 cells with different concentrations. As a control, the enhanced green fluorescent protein (EGFP) gene in pcDNA3.2 was deployed to transfect CAL27 cells. After co-culture with lentivirus particles from supernatant of 293T cells for 72h, the transfected CAL27 cells were collected and examined for the EGFR expressions by western blot assay.

**Western Blot**

Proteins from EGFR-overexpressed CAL27, and corresponding vector cells, also the MVs from the cells were collected. In addition, 20 μg protein of Annexin V^-^ sorted or unsorted MVs were prepared. The protein concentrations were calculated by bicinchoninic acid assay (Pierce BCA, Thermo Fisher Scientific, USA). Proteins were denatured by adding 5× sodium dodecyl sulfate–polyacrylamide gel electrophoresis (SDS-PAGE) buffer (Servicebio, Wuhan, China) and transferred in PVDF (polyvinylidene fluoride) membrane. The transferred membranes was incubated with EGFR (1:1000, Cell signaling technology), CD8 (1:1000, Proteintech), AXL (1:1000, Cell signaling technology), and GAPDH (1:1000, Proteintech) overnight at 4°C. Then the membranes were incubated with the corresponding second antibodies for 1 h and washed by TBST (0.1% Tween in TBS). Finally, the membranes were incubated with ECL mix (prepared by ECL A and ECL B) for 2 min and visualize the result in the dark room.

**Liquid chromatography (LC)-electrospray ionization (ESI) tandem mass-spectroscopy (MS/MS) Analysis**

After characterization, a total of 50 μg of protein was used for proteomic analysis using for each of the groups (n=2, for CAL27 Vector and CAL27 EGFR-OE derived MVs, respectively). The following LC-MS analysis was performed by Wuhan Servicebio Technology Co., Ltd, Wuhan, China. Briefly, we use trypsin to digest the precipitated MV samples. The resulting peptides were analyzed on an Ultimate 3000 RSLCnano-UHPLC system connected to a Q Exactive mass spectrometer (Thermo Fisher Scientific, Bremen, Germany) equipped with a nano-electrospray ion source. The reference database (SwissProt database, Human) was used for peptide identification and the multiple identified. Protein was identified and validated, using Scaffold_4.8.7 (Proteome Software Inc).

**MVs uptake assay**

Normal oral epithelial cells were stained with the CellTrace™ CFSE Cell Proliferation Kit (Invitrogen, USA) according to the manufacturer’s instructions. The PKH67 Fluorescent Cell Linker Kit (Sigma) was used to label CAL27 cell derived MVs. PKH67 dye solution (1:1000) was mixed with 10 μg or 50 μg MVs for 20 min, washed with PBS, and centrifuged at 20,000 x g at 4°C for 60 min. Then, PKH67-labeled MVs were added to the DIL-labeled cells and co-cultured for 24 h. Uptake of MVs at different time points was observed and captured by a confocal fluorescence microscope and flow cytometry.

**Wound healing assay**

For wound-healing experiment, cells grew to 80% confluence in six-well plates and were scratched by sterile 200 µL pipette tips. The wound closure was observed and was imaged under a microscope at 0 and 48 h after MVs treatment (20 μg/ml). The area of un-adherent or total area was calculated by Image J software and compared by GraphPad software.

**Immunohistochemistry**

OSCC tissue from xenograft tumor models were collected and formalin-fixed for immunohistochemistry. Briefly, tissue sections were deparaffinized using xylene and rehydrated in PBS. Citrate solution (Servicebio, Wuhan, China) was used for antigen retrieval. The slides contained citrate solution was heated to 125 °C and sustained for 8 min and then slides were cooling to 90 °C for 1 min. Then the slides were carried out in the room temperature for 2 h. 3% hydrogen peroxide was incubated with slides for 20 min. Goat serum (Zhongshan golden bridge, Beijing, China) was used for non-specific binding blocking for 10 min. EGFR antibody at a dilution of 1:200 (Cell signaling technology, 1:200) were incubated for slides overnight at 4 °C. After washing in PBS, the secondary antibody (Abbkine, goat-anti-rabbit, 1:200) was incubated with the slides for 15 min at room temperature. The slides were incubated with DAB for 30 s. Finally, slides were stained with hematoxylin for visualization. Then slides were dehydrated using ethanol and cover slipped by thin glass for detection.
